# Effects of solution conditions on the self-assembly of the chaperone protein DNAJB6b

**DOI:** 10.1038/s42004-025-01697-7

**Published:** 2025-10-01

**Authors:** Andreas Carlsson, Victoria Maier, Celia Fricke, Tinna Pálmadóttir, Ingemar André, Ulf Olsson, Sara Linse

**Affiliations:** 1 Biochemistry and Structural Biology, Department of Chemistry, Lund University, SE-221 00 Lund, Sweden; 2 Center for Functional Protein Assemblies and Department of Bioscience, TUM School of Natural Sciences, Technical University of Munich (TUM), 85748 Garching, Germany; 3 Department of Biotechnology and Biomedicine, Technical University of Denmark, 2800 Kongens Lyngby, Denmark; 4 Division of Physical Chemistry, Department of Chemistry, Lund University, SE-221 00 Lund, Sweden

**Keywords:** Chaperones, Biophysical chemistry, Protein aggregation

## Abstract

Chaperone proteins are essential for maintaining proteostasis. Their main role is to assist with the folding of other proteins and to prevent the aggregation of misfolded proteins. The molecular chaperone DNAJB6b efficiently suppresses amyloid formation of several peptides. This activity may rely on the same physicochemical properties as those driving chaperone self-assembly into large micellar-like oligomers. We have therefore undertaken a systematic study of DNAJB6b’s self-assembly under different solution conditions. Using complementary biophysical techniques, we probe variations in aggregation number distribution and hydrodynamic radius, upon variation of pH, temperature, ionic strength, or anions across the Hofmeister series. We find that DNAJB6b maintains its propensity to self-assemble under all solution conditions examined. The size and compactness of the micelles change upon unfolding of the C-terminal domain, although a folded C-terminal domain does not drive micelle formation, which can likely be ascribed to hydrophobic interactions in the linker region. Mass photometry reveals that monomers of DNAJB6b coexist at equilibrium with the micelles. Furthermore, the free energy barrier for micelle dissociation into monomers was estimated by measuring dissociation rate constants at different temperatures.

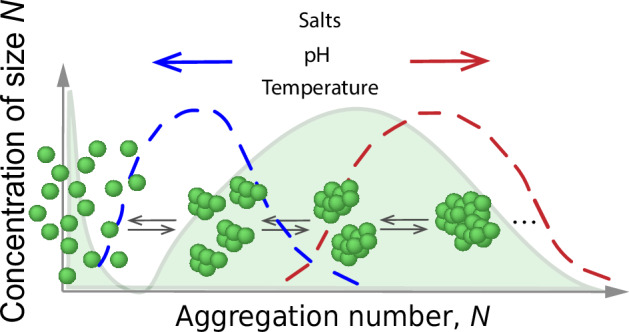

## Abstract (web summary)

The ability of the molecular chaperone DNAJB6b to suppress amyloid formation may be linked to its propensity to self-assemble into large micellar-like oligomers, but systematic studies of its solution-phase behaviour are lacking. Here, using a combination of mass photometry and microfluidic diffusional sizing, the authors probe variations in aggregation number distribution and hydrodynamic radius as a function of pH, temperature, ionic strength, or the nature of the anion across the Hofmeister series.

## Introduction

DNAJB6b (hereafter called JB6) is a remarkably efficient amyloid suppressor, which is part of the endogenous defense against protein misfolding diseases. JB6 prevents against amyloid formation of for example *α*-synuclein (involved in Parkinson’s disease, Lewy body dementia, and multiple system atrophy)^1–3^, polyglutamine peptides (Huntington’s disease)^4–9^, amyloid *β* peptides (Alzheimer’s disease)^10–12^, IAPP (diabetes type 2)^13^, and TDP-43 (amyotrophic lateral sclerosis)^14^. JB6 thus combines a high specificity in action with a low specificity in terms of target sequence.

JB6 contains a 110-residue low-complexity region between two folded domains. Both the C-terminal domain, CTD, of JB6^15^ and part of the linker region have been reported to be important in interactions with amyloids^7,11^, whereas the J-domain (N-terminal domain, NTD) interacts with HSP70^5,16,17^.

A particular property that JB6 shares with several other chaperone proteins^5,18–20^, is its propensity to self-assemble in solution into large micellar aggregates^4,7,21,22^. We intentionally use the concept of micelles^23^ to emphasize two critical features: these aggregates are of finite size and they correspond to a thermodynamic equilibrium state, characterized by a defined size distribution^23^. Notably, the idea of protein micelles was already proposed in the 1980s by Augusteyn and Koretz in their studies of *α*-crystallin aggregates in the eye lens^24^. The formation of finite size equilibrium micelles is fundamentally different from the precipitation of e.g. practically infinite amyloid fibrils. While amyloid formation can be described as a phase separation^25^, micelle formation does not involve a phase transition. In the case of precipitation, there is a saturation concentration, or solubility, corresponding to a phase boundary, above which precipitation occurs. The solubility, being an important characteristic property of amyloid proteins, has been determined for amyloid-*β*(1–40)^26,27^ and *α*-synuclein^28^. The corresponding property of micelle-forming compounds is the critical micelle concentration, cmc, which can be viewed as a kind of solubility. The cmc of JB6 was recently measured to be 120 nM at 22 °C and pH 8.0^22^. This low cmc may be linked to the strong propensity of JB6 subunits to suppress amyloid formation^29^, which has been inferred to rely on binding to oligomeric and fibrillar rather than monomeric client species^10–12^. Although the molecular mechanism behind this suppression is not fully understood, short-range hydrophobic interactions are likely to play a key role. This is in line with the fact that JB6 is able to suppress amyloid formation of many types of clients, indicative of non-specific driving interactions. The exposure of hydrophobic residues implies that a highly potent chaperone is expected to be only weakly water soluble, to favor the interactions between chaperone and client. This property of the chaperone also makes it prone to self-assemble. With the formation of relatively small, non-toxic micelles - as opposed to large precipitates—higher concentrations of the chaperone can likely be present without being harmful to the cellular environment.

Given a close connection between JB6’s efficiency in acting as a chaperone and its ability to self-assemble^29^, it follows that a detailed characterization and understanding of the self-assembly may help to interpret and understand chaperone action on a molecular and thermodynamic level. In fact, the multifaceted chaperone interactions, with itself or with clients, may rely on the same protein properties that give the isolated JB6 subunits a relatively poor aqueous solubility, reflecting a high chemical potential in solution^30^. The JB6 self-assembly is highly challenging to analyze in vivo due to multiple competing equilibria. Through studies of the chaperone self-assembly in vitro, including both kinetics and equilibrium at a variety of solution conditions, it is possible to unravel the driving forces for this process. Considering that the chaperone subunits are the active species, this provides highly valuable insight into the concentration of active chaperone species and how this is controlled by the solution conditions. In this work, we have therefore undertaken a detailed quantitative characterization and analysis of JB6 self-assembly in solution and how it depends on temperature, pH and ionic strength, including dependence along the Hofmeister series, where the latter can provide information about the role of hydrophobic interactions^31,32^.

## Results

### Equilibrium size distribution by mass photometry

**Fig. 1:**
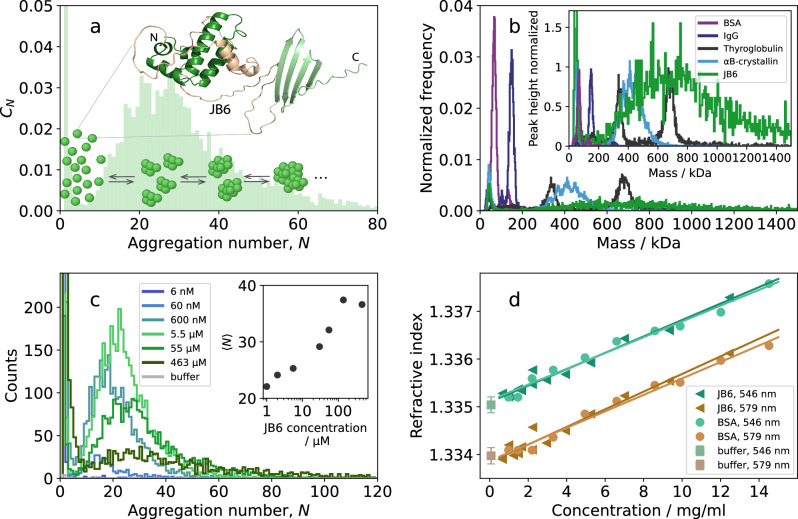
Using mass photometry to examine the equilibrium size distribution of JB6. **a** Schematic illustration of the equilibrium size distribution of JB6, where *C*_*N*_ is the concentration of an *N*-mer. The structure of a JB6 monomer is given as an AlphaFold2 prediction with the NTD in dark green, the CTD in light green, and the linker in gold. **b** Mass photometry data of the size distributions of five different proteins, normalized to either area or peak height (inset). BSA (66 kDa), IgG (150 kDa), and thyroglobulin (669 kDa) were used as standard proteins with known masses (see Supplementary Note 1, Fig. S1). *α*B-crystallin (20.2 kDa) self-assembles with a narrow size distribution, in contrast to the very broad distribution of JB6 (55 μM). **c** The concentration dependence of JB6 size distribution. The inset shows how the average *N* of the micelles, at concentrations above 1 μM, changes with JB6 concentration. Not all of the measured concentrations are shown as distributions, to avoid cluttering of the graph. **d** Refractive index measurements of JB6 and BSA. To trust the masses given by mass photometry, the refractive index dependence of protein concentration, *dn*/*dc*, should be similar, which they are (0.17 ml/g for both JB6 and BSA at 546 nm, and 0.18 ml/g and 0.17 ml/g, respectively, at 579 nm).

The broad size distribution observed for JB6^4,21,22^ implicates that there is no strongly preferred aggregation number, *N*, and micelles of many different sizes co-exist at equilibrium. This is illustrated schematically in Fig. 1a, where the concentration of an *N*-mer is denoted *C*_*N*_, and an AlphaFold2 structure prediction of the JB6 monomer is included^33,34^. Here, mass photometry^35^ was used to measure the equilibrium size distribution of JB6, at pH 8.0, 20 mM sodium phosphate buffer (NaP), and 0.2 mM EDTA, at ambient (room) temperature. Scattering intensities of single particles are converted to particle masses using a calibration with standard proteins of known masses. In this work, bovine serum albumin, BSA (66 kDa), human immunoglobulin G, IgG (150 kDa), and bovine thyroglobulin (669 kDa) were used (Fig. 1b, area respectively peak height normalization). The calibration is further detailed in Supplementary Note 1, Fig. S1. The size distribution of JB6 is indeed very broad, as seen in the comparison to the self-assembling chaperone protein *α*B-crystallin, which forms essentially monodisperse aggregates. The width of the 669 kDa thyroglobulin peak reflects the mass uncertainty at this particle size. The full width at half-maximum is found to be *σ* ∗ 2.355 = 52 kDa, similar to findings in^36^. The self-assembly has been shown to be concentration dependent, with a cmc of about 120 nM^22^. In Fig. 1c the size distributions at different JB6 concentrations are presented and the inset shows the average aggregation number, 〈*N*〉, for concentrations above 1 μM, which we find increases with increasing JB6 concentration. Samples with concentrations greater than 200 nM were diluted to 200 nM less than a minute before measurements. With 〈*N*〉 > 20, the particle concentration is around 10 nM, allowing for single particle analysis. The measured size distribution can be considered representative of the equilibrium at the incubated concentration since the dissociation of the micelles occurs on a time scale of tens of hours at room temperature (see below, Fig. 4), while each measurement takes 60 s.

Because the polarizability differs very little between proteins^36^, the signal intensity in mass photometry is, to a good approximation, directly proportional to the protein molar mass. To confirm that this also holds for the JB6 assemblies, we determined the refractive index increment (*dn*/*dc*) for JB6 solutions. *dn*/*dc* is proportional to the polarizability of particles^37^. The refractive index at 20 °C was measured at two wavelengths, 546 and 579 nm, as a function of protein mass concentration (Fig. 1d). For comparison, measurements were also performed with BSA. A linear fit to each concentration series provides a slope corresponding to *dn*/*dc*. With *dn*/*dc* of 0.17 ml/g at 546 nm for both proteins and 0.18 and 0.17 ml/g at 579 nm for JB6 and BSA, respectively. We conclude that *dn*/*dc* is essentially the same for the two proteins.

### The JB6 subunit

**Fig. 2:**
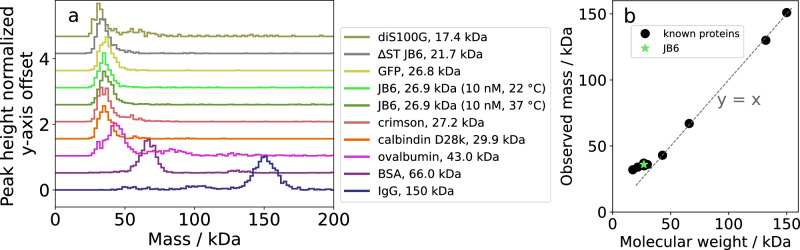
Pushing the limits of mass photometry to study the JB6 subunit. **a** Mass histograms of eight proteins with known masses, and 10 nM pre-equilibrated JB6 at room temperature and 37 °C. **b** The obtained masses from (**a**) are plotted versus each molecular weight (with both the monomer and dimer of BSA). JB6 aligns with other proteins with approximately monomeric masses of 26.9 kDa, with no observable dimerization.

An important question concerns whether JB6 exists as a monomer or dimer together with the micelles. Several J-domain proteins dimerize via the common dimerization domain^16,38–40^, but JB6 has eluded a clear characterization and lacks this dimerization domain. The 26.9 kDa JB6 monomer is slightly below the lower limit of protein mass that is considered possible to be correctly determined by the mass photometer^41^. However, a 53.8 kDa dimer is above this limit. In order to investigate whether the JB6 subunit is monomeric or dimeric, eight proteins with known masses were measured with mass photometry to produce a calibration curve in the mass range 17.4−150 kDa (Fig. 2a). The obtained masses are plotted versus each nominal molecular weight in Fig. 2b. A 1:1 linear regime is observed above ca 40 kDa, while linear extrapolation from this regime overestimates the mass below 30 kDa. In Fig. 2A we also show the results for 10 nM JB6 (<cmc), measured at room temperature and 37 °C, respectively. From comparing with the calibration proteins it is clear that the data for JB6 are consistent with the protein being in a monomeric state. No dimer peak is observed. This is also the case at higher JB6 concentrations (> cmc) and in 150 mM NaCl, pH 7.4 (Supplementary Note 4, Fig. S4).

### Comparing size distributions at room temperature and 37 °C

**Fig. 3:**
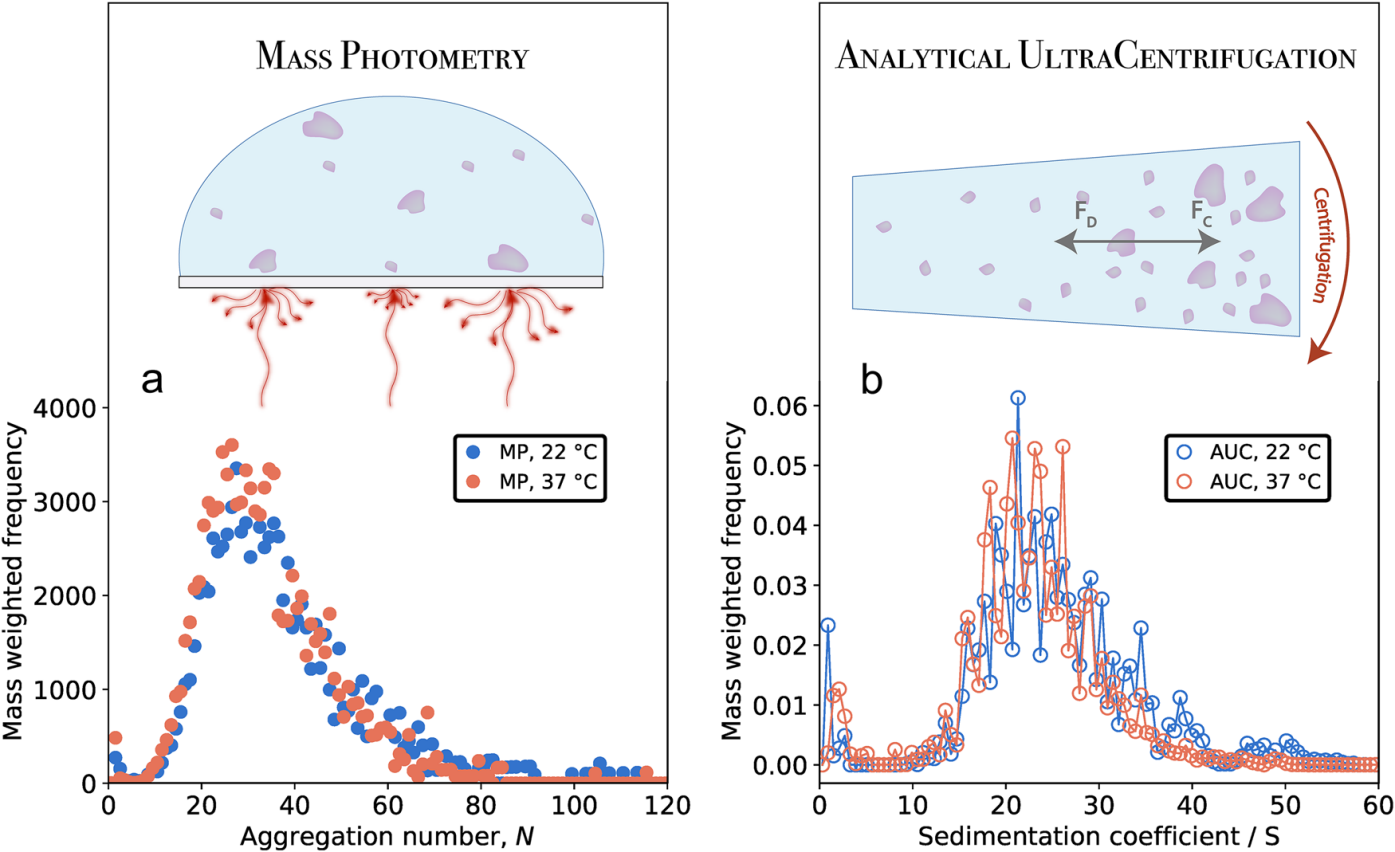
Comparison of the equilibrium size distribution of 30 μM JB6 at 22 °C (blue) and 37 °C (red). **a** Mass photometry data (mass-weighted distributions) and a schematic illustration of the technique principle. Light is scattered more or less by single particles close to the surface, depending on their respective masses. **b** The same samples as in (**b**) analyzed with AUC in velocity sedimentation mode. Plotted are the temperature corrected sedimentation coefficients of 20 °C. The schematic illustrates how this technique relies on the detection of sedimentation velocity during centrifugation, depending on both the centrifugal force, F_C_, and the drag force, F_D_.

**Fig. 4:**
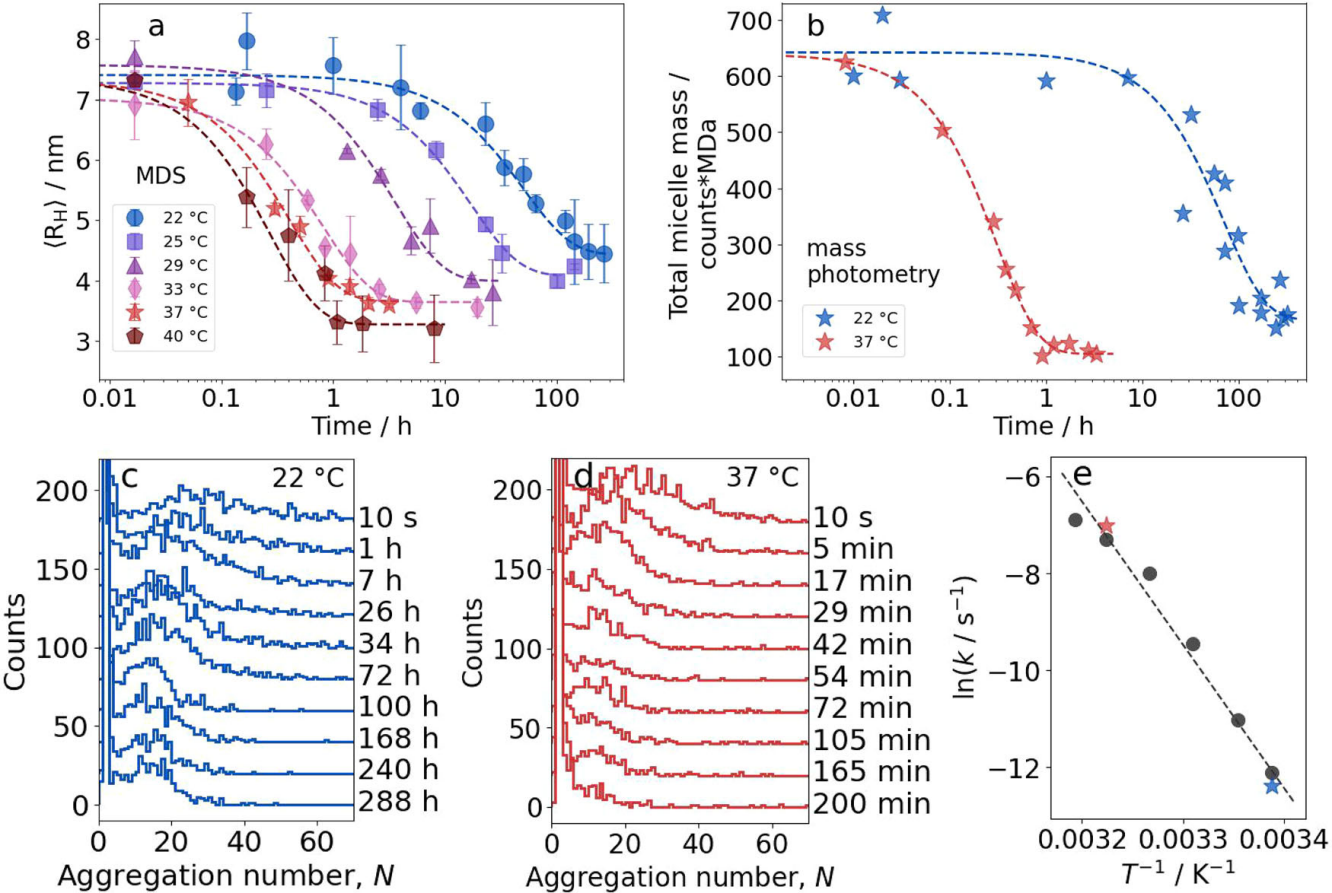
Micelle dissociation of JB6 at different temperatures. **a** 〈R_H_〉, measured with microfluidic diffusional sizing, MDS, as a function of time since dilution, at temperatures (22−40 °C) as indicated by color and symbol in the legend. The error bars represent the standard deviation of three technical replicates. Each series was fitted to a single exponential decay function, ${\langle {{{{\rm{R}}}}}_{{{{\rm{H}}}}}\rangle }_{(t)}=a* {{{{\rm{e}}}}}^{-kt}+c$, shown as dashed lines. **b** Dissociation kinetics at 22 and 37 °C, probed by the change of total micelle mass during 60 s data collection with mass photometry. 34 μM non-labeled JB6 was diluted to 50 nM at time zero. **c**, **d** The change in mass distribution upon dilution at 22 respectively 37 °C. The distributions are plotted with shifted y-axis to allow for better comparison. **e** Arrhenius plot of the obtained dissociation rate constants (from MDS in black circles and from mass photometry in colored stars) versus the inverse of the temperature. The fitted line describes a thermally activated dissociation process, ${k}_{(T)}=A* {{{{\rm{e}}}}}^{-{E}_{a}/RT}$, where the activation energy, *E*_*a*_, is found to be 250 kJ/mol.

To learn about the temperature dependence of the JB6 self-assembly, the size distributions of 30 μM JB6 were compared at room temperature (≈22 °C) and 37 °C (pH 8.0, 20 mM NaP, and 0.2 mM EDTA). The distributions were measured using both mass photometry (Fig. 3a) and AUC, in sedimentation velocity mode (Fig. 3b). As can be seen, the size distributions are essentially identical at the two temperatures. This is confirmed by both techniques. The temperature-corrected sedimentation coefficient of 20 °C is consistently used and displayed. The mass photometer operates at ambient temperature, but the measurement time is short (1 min) compared to the time scale of micelle dissociation (Fig. 4), and the obtained size distribution can be considered representative of the distribution at the incubation temperature. The raw data distributions of the mass photometer (counts for a certain particle mass) are here multiplied by *N*, to obtain a mass-weighted distribution (Supplementary Note 5, Fig. S5), allowing for comparison to the AUC data, which are based on absorbance measurements and therefore are mass-weighted. We note that the monomer concentration remains low at both temperatures, in line with the similar size distributions.

### The kinetics of micelle dissociation

The kinetics of JB6 micelle dissolution was investigated at different temperatures by measuring the average hydrodynamic radius 〈R_H_〉 as a function of time, *t*, after a step dilution from 5.8 μM to 55 nM in time resolved microfluidic diffusional sizing (MDS) experiments. The results are presented in Fig. 4a. The 〈R_H_〉 was found to decrease as a function of time and plateau at slightly different sizes, 3.3 ± 0.4 nm at 40 °C and 4.5 ± 0.5 nm at 22 °C. Fits to single exponential decay functions, 〈*R*_*H*_〉(*t*) = *ae*^−*kt*^ + *c*, describe the data well, indicative of a single rate-limiting step in the dissociation process. The dissociation rate constant, *k*, and constants *a* and *c*, are fitted parameters at each temperature. To complement the MDS data with another technique and with non-labeled protein, the mass photometer was utilized to measure how the mass distribution changes as a function of time after dilution, at 22 respectively 37 °C (Fig. 4b–d). The total mass of all observed micelles during 60 s of data collection is used as a reporter on the micelle dissociation. This is calculated as the sum of each bin count multiplied by its bin mass. Furthermore, the mass photometry data complement the MDS data with information on the aggregation state at equilibrium. By comparing the distributions at 288 h at 22 °C and 200 min at 37 °C, it is clear that more micelles are kept from dissociating at 22 °C compared to at 37 °C. This explains the difference in 〈R_H_〉 at the final stages.

Using the obtained dissociation rate constants from both MDS and mass photometry, it is possible to estimate the activation energy, *E*_*a*_, of the thermally activated dissociation process by an Arrhenius analysis (Fig. 4e), $k(T)=A{{{{\rm{e}}}}}^{-{E}_{a}/RT}$, where *A* is a fitted coefficient. The activation energy of the dissociation event is estimated to *E*_*a*_ = 250 kJ/mol.

### pH dependence

**Fig. 5:**
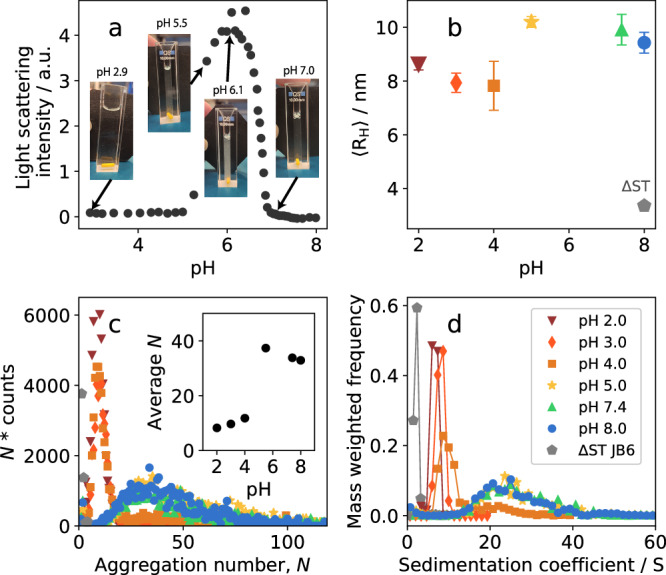
pH dependence of the self-assembly of JB6. **a** Light scattering intensity (here used as a turbidity reporter) of 10 μM JB6 as a function of pH. Titration with 20 mM phosphoric acid to a starting solution with pH 8.0, 20 mM NaP, 0.2 mM EDTA, at room temperature. **b** 〈R_H_〉 of 30 μM JB6 in 20 mM NaP at room temperature, measured with MDS at different pH values. Standard deviation of three technical replicates. The 〈R_H_〉 of a mainly monomeric mutant of JB6, denoted as ΔST, is shown in a gray pentagon as comparison. **c** Mass-weighted size distributions at pH 2-8, obtained with mass photometry of the same samples as in (**b**). Inset with average *N* of each mass distribution. **d** Distributions in sedimentation coefficients obtained by AUC of the same samples as in (**b**) together with the ΔST JB6 mutant. Each distribution is given by solutions to a Monte Carlo simulation that describes the noise reduced raw data absorbance scans, obtained in velocity sedimentation mode. The solutions are connected by a line to help distinguishing each pH series. The color and symbol representation in the legend are valid for panel (**b**−**d**).

To determine the phase boundaries of JB6 precipitation, with respect to pH, light scattering was used to probe the turbidity of 10 μM JB6. The temperature was kept constant (20 °C) and the ionic components were similar throughout the titration by stepwise addition of 20 mM phosphoric acid to the initial 20 mM NaP buffer, 0.2 mM EDTA, pH 8.0. JB6 is soluble below pH 5 and above pH 7, but precipitates in between (Fig. 5a). This is visually supported by pictures of the cuvette, where the sample has a clear appearance at pH 6.9 and 2.9, but cloudy at pH 6.1 and 5.5. The result is consistent with previous reports^28,42^, with the plausible explanation that it is primarily the net charge of the linker that drives the precipitation^42^.

In the pH regions in which JB6 remains in solution (below pH 5 and above pH 7), MDS was used to measure the 〈R_H_〉 of 30 μM JB6, with 20 mM NaP, 0.2 mM EDTA, at room temperature. The pH values examined are 2.0, 3.0, 4.0, 5.0, 7.4, 8.0. To compare the 〈R_H_〉 with a reportedly mainly monomeric variant of JB6^21^, the 〈R_H_〉 of the deletion mutant ΔST (residue 132-184 deleted, see Supplementary Note 7, Fig. S7 for more information), 3.3 nm, is shown as a gray pentagon. The 〈R_H_〉 of JB6 wt is much larger than expected for monomers at all pH values, ranging from about 8 nm at pH 4.0 to ~10 nm at pH 5.0.

The same samples used in MDS measurements were also analyzed by mass photometry and AUC (Fig. 5c, d) to obtain equilibrium size distributions in both mass and sedimentation coefficients at each pH value. The two techniques provide highly similar distributions and reveal a strong pH dependence of the JB6 self-assembly. At pH 5.0 and 7.4, a similar distribution to that at pH 8.0 is observed. However, at pH 2.0 and 3.0, the distribution is narrower and 〈*N*〉 ≈ 10. At pH 4.0, mainly the peak corresponding to the smaller masses is visible, but there is also a small peak at higher 〈*N*〉 ≈ 40, implying a bimodal distribution. The variation of 〈*N*〉 with pH is plotted in the inset of Fig. 5c. As can be seen, there is a sharp change between pH 4 and 5.

Regarding the monomer concentration, no change is observed with varying pH. If the monomer concentration would have been dramatically higher at any pH, this would have been distinguished. It is also noted that the ΔST mutant behaves as expected for a monomeric, globularly folded protein of 21.7 kDa, both in AUC and mass photometry.

**Fig. 6:**
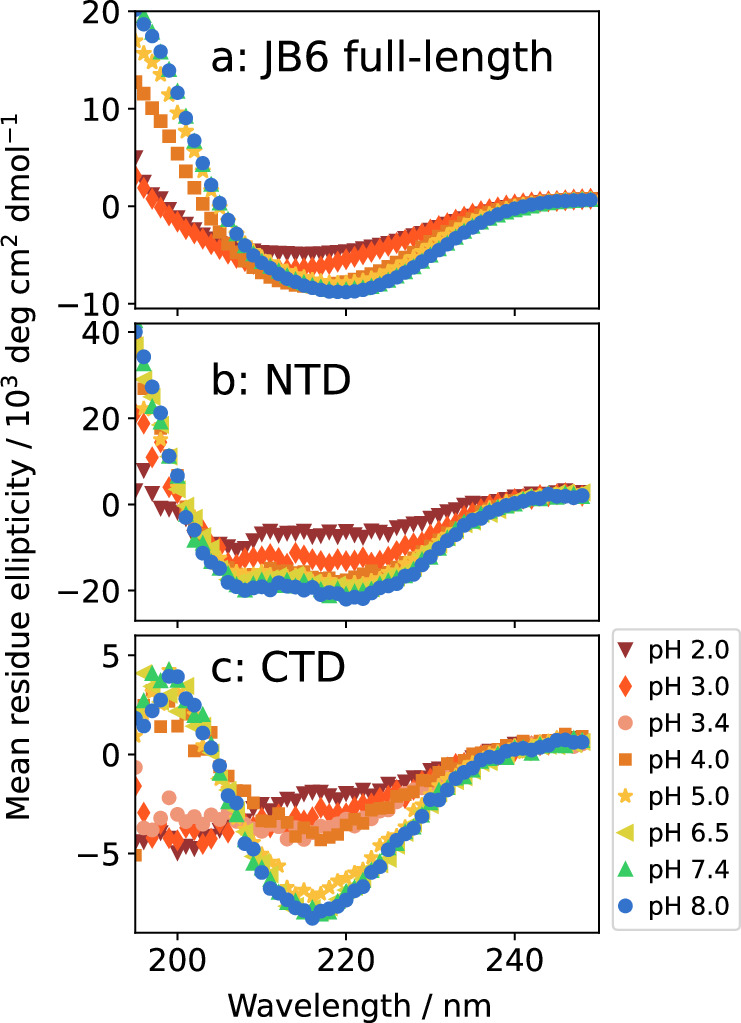
Circular dichroism spectra at different pH values. The analyzed samples are (**a**) JB6, (**b**) NTD, (**c**) CTD, at 5 μM protein in 20 NaP, 0.2 mM EDTA, room temperature.

Circular dichroism (CD) spectroscopy was performed (Fig. 6) to investigate the level of folding at different pH values and potentially shed light on the sharp change in the size distribution between pH 4 and 5, as observed in Fig. 5c-d. In addition to full-length JB6, the isolated NTD (residue numbers 1-71) and CTD (residue numbers 186-241) were also measured. At pH 5-8, there are no or little changes in the spectra; the NTD displays typical *α*-helical spectra and the CTD spectra imply a mostly *β*-sheet structure, in line with the literature^21,42^. However, the CTD loses most of its signal intensity around pH 4, indicative of loss of its folded structure, and appears even less folded below this pH. This clearly correlates with the abrupt change in size distributions. The NTD maintains its spectrum, and thereby its fold, at the lower pH values, but displays reduced secondary structure at pH 2.

### Combining MDS, AUC and mass photometry

To this point, in addition to the level of folding at each pH value, we have obtained information on the size distribution of JB6, expressed in terms of different physical properties due to the nature of the used techniques; MDS provided an average R_H_, mass photometry gave a size distribution in particle masses, and AUC gave sedimentation coefficients as output. We will now attempt to combine and compare the data from the three techniques. The Svedberg equation (Eq. (1)) relates the particle mass with its sedimentation coefficient and R_H_:

1 $$S=\frac{{m}_{{{{\rm{p}}}}}-{m}_{{{{\rm{w}}}}}}{6\pi \eta {{{{\rm{R}}}}}_{{{{\rm{H}}}}}}=\frac{{V}_{{{{\rm{p}}}}}{\rho }_{{{{\rm{p}}}}}-{V}_{{{{\rm{p}}}}}{\rho }_{{{{\rm{w}}}}}}{6\pi \eta {{{{\rm{R}}}}}_{{{{\rm{H}}}}}}=\frac{{m}_{{{{\rm{p}}}}}\left(1-\frac{{\rho }_{{{{\rm{w}}}}}}{{\rho }_{{{{\rm{p}}}}}}\right)}{6\pi \eta {{{{\rm{R}}}}}_{{{{\rm{H}}}}}}$$

**Fig. 7:**
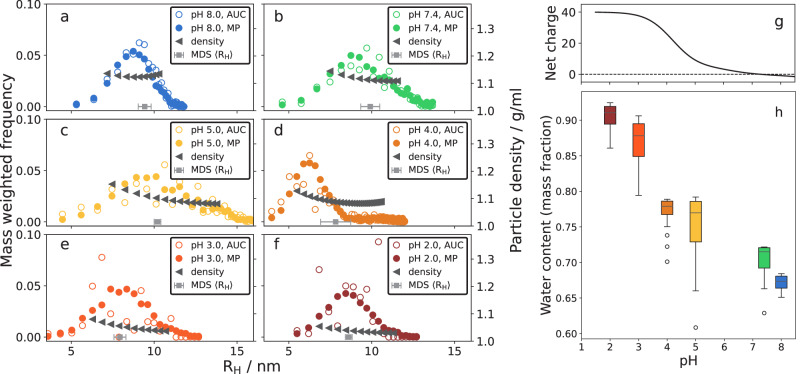
Combining distributions from mass photometry and AUC to obtain information of the packing densities of the particles. **a**−**f** Alignment of mass-weighted mass photometry data (filled circles) and AUC data (open circles, plotted as a function of R_H_). Particle densities, here defined as the particle mass divided by the volume of a sphere with radius R_H_, are plotted relative to the right *y*-axis. The gray square and error bar close to the x-axis indicates the 〈R_H_〉 and standard deviation at each pH, obtained with MDS (same data as in Fig. 5b). **g** Predicted net charge of JB6 as a function of pH, calculated with model pKa values from^46^. **h** The mass fraction of water of the particles for each pH, given by the particle densities in (**a**−**f**).

*S* is the sedimentation coefficient, *m*_p_ the particle protein mass, *m*_w_ the mass of excluded water, *η* the solvent viscosity, R_H_ the hydrodynamic radius, *V*_p_ the protein volume, *ρ*_p_ the density of pure protein: 1.39 g/ml (mean value from findings in refs. ^43^ and^44^), and *ρ*_w_ is the density of water, 1 g/ml. By aligning the mass and sedimentation coefficient distributions, binned to obtain a mass for each sedimentation coefficient, an R_H_ at each point can be calculated using the Svedberg equation (Fig. 7a–f). To compare the distributions, they should be weighted in the same way. AUC is based on absorbance and is thus mass weighted. Hence, the mass-photometry distributions are multiplied with *N* to also be mass weighted.

The 〈R_H_〉 from MDS (same data as in Fig. 5b) is plotted as gray squares and error bars close to each x-axis, for comparison. The orthogonal methods to obtain R_H_ are thus in good agreement. As a measure of the micelle packing density, we use the ratio between particle mass and the spherical volume defined by the R_H_, providing a particle density which is plotted as black triangles versus R_H_ and relative to the right y-axis. By relating the particle densities to the densities of water (1 g/ml) and pure protein (1.39 g/ml), the relative amounts of water and protein in the spherical volume, defined by R_H_, can be estimated (Fig. 7h). These values vary from around 65 % water at pH 8.0 to around 90 % at pH 2.0. It may be noted that proteins usually self-assemble with 26−90 % solvent content in the crystalline phase^45^. The water content can be related to the change in the predicted net charge of JB6 as a function of pH, Fig. 7g, using model pKa values^46^.

### Effects of ionic strength and the anion Hofmeister series

**Fig. 8:**
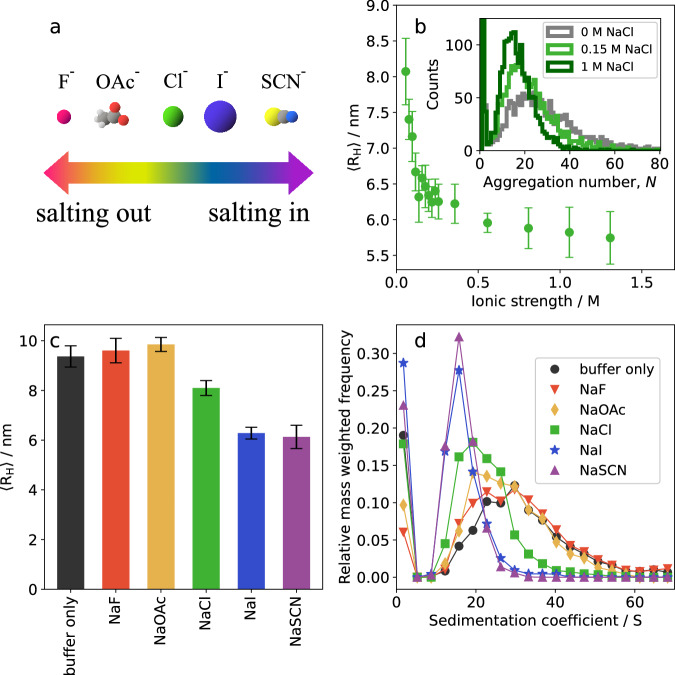
Dependence of added salts. **a** Representation of the anions used, ordered according to the Hofmeister series. **b** 5 μM JB6 in 20 mM NaP, pH 8.0. 〈R_H_〉 from MDS as a function of ionic strength (NaCl concentration). **c** 〈R_H_〉, from MDS, of 30 μM JB6 in 20 mM NaP, 0.2 mM EDTA, 150 mM of each salt in the Hofmeister series, pH 8.0, at room temperature. A sample without any added salt (buffer only) is included in the study for comparison. Four replicates of each condition, with standard deviation as error bars. **d** Distribution of sedimentation coefficients of the same samples as in (**c**) color and symbol coded as described in the legend.

At pH 8, JB6 has a weak negative net charge, and one may expect to see specific ion effects^47,48^. Different sodium salts, having different monovalent anions across the Hofmeister series (Fig. 8a),^49^ ranging from typical “salting out” to typical “salting in”, were added to JB6 solutions (20 mM NaP, 0.2 mM EDTA, pH 8.0, room temperature), to investigate possible effects of anion binding^32,50^. NaCl is a commonly used salt for adjusting the solution’s ionic strength, and was used in a first set of experiments to characterize, using a combination of MDS and mass photometry, the effect of salt on the self-assembly of 5 μM JB6, over a large range of salt concentrations. As can be seen in Fig. 8b, 〈R_H_〉 initially decreases strongly with increasing concentrations of NaCl, to level off at a value of  ≈ 6 nm above a NaCl concentration of 0.5 M. Mass photometry data (Fig. 8b, inset) confirm that the aggregate size distribution shifts to lower aggregation numbers with increasing ionic strength. We note that the micelles remain stable at 1 M salt where the Debye screening length is ~0.3 nm. This implies that chloride has a salting in effect here.

In a second set of experiments, the JB6 concentration was kept constant at 30 μM, and the effects on the micelle size distribution of different monovalent anions, at a fixed concentration of 150 mM, were compared. The 20 mM NaP buffer alone has at pH 8 an ionic strength of ~50 mM. The salts were chosen to keep the ionic strength and valency constant, and to cover the range from “salting out” to “salting in” anions (Fig. 8a). In Fig. 8c we compare the specific ion effects on 〈R_H_〉, measured by MDS. Compared to buffer only, where 〈R_H_〉 ≈ 9 nm, the effects of the typical salting out anions fluoride and acetate are essentially negligible. However in the case of chloride, 〈R_H_〉 has dropped to 8 nm, and for iodine and thiocyonate 〈R_H_〉 ≈ 6 nm. This change in average micelle size is confirmed by the AUC data presented in Fig. 8d, as the distribution of sedimentation coefficients. Again, in the case of fluoride and acetate, there is essentially no difference compared to buffer only. However, for chloride, a minor shift to smaller micelle sizes is observed, and for iodine and thiocyanate there is a large decrease in the average size and a narrowing of the distribution. Furthermore, in the AUC data we note that the monomer concentration follows the expected trend of the Hofmeister series, with higher monomer concentration in the presence of salting in-ions. Notably, at the commonly used NaCl concentration of 150 mM in experiments in vitro (phosphate-buffered saline), the size distribution is shifted towards smaller micelles, compared to without NaCl. This salting-in effect probably needs to be taken into account when comparing chaperone activity at different NaCl salinities.

## Discussion

The ability of JB6 to self-assemble is most likely coupled to its chaperone function with both properties governed by similar hydrophobic interactions. Therefore, a detailed characterization of the self-assembly behavior of JB6 under different solution conditions has been carried out in the present study. JB6 is involved with complex systems such as the HSP70-machinery and amyloid formation. Adding a half-folded and surface-active chaperone that self-assembles in a concentration-dependent manner with a heterogeneous size distribution, adds another complexity layer. Quantitative analyses of mixed systems will be greatly facilitated by the detailed knowledge of the self-assembly equilibrium and subunit dynamics of JB6 alone. We utilize here a biophysical approach to characterize the JB6 self-assembly process in terms of its response to changes in solution conditions with an aim to understand which types of interactions govern the self-assembly.

To enable a thorough analysis, we use both mass photometry and AUC to obtain size distributions based on different particle properties; mass in the case of mass photometry and mass together with hydrodynamic radius for AUC. Each technique has its advantages and disadvantages, and by combining these methods, we can learn more about the self-assembly characteristics. AUC is a classical tool to examine the oligomeric states of proteins. It provides information about the entire solution at the temperature of choice (4−40 °C)^51^. However, the main output is a distribution of sedimentation coefficients. To convert this to a distribution of masses, knowledge of hydrodynamic properties is required, as well as further data treatment and fitting. Mass photometry is a relatively new technique that can provide the mass distribution of a sample using single-particle measurements based on the light scattering of the particle at a surface and at ambient temperature^36^. The lower limit of reliable quantification of the particle mass is ~40 kDa^41^, which is supported by our results (Fig. 2b). The JB6 monomer has a mass of 26.9 kDa, and even though we show that some information can be obtained of masses below 40 kDa, the precise mass and number of counts are less quantifiable. However, the analysis of several proteins with known masses below 40 kDa, together with no observable JB6 dimer peak, enables us to conclude that the JB6 subunit, which is in equilibrium with the larger micelles, is monomeric and not dimeric.

In order to understand, on the molecular level, how JB6 suppresses amyloid formation, the question of the dominating JB6 subunit is important. In Fig. 2, we show by direct mass photometry measurement that the subunit coexisting with the larger micelles is monomeric. There is no sign of dimers in the 34 μM sample (Supplementary Note 4, Fig. S4, in SI). In these experiments, samples were rapidly diluted to 200 nM before the measurements. The larger micelles dissociate slowly, and the measured micellar size distribution accurately represents that of the initial high concentration state. Regarding the question whether also the observed monomer state is a true representation of higher concentrations, or if there has been a rapid dissociation of dimers, we may consider the monomer-dimer equilibrium described by the association constant ${K}_{2}={c}_{2}/{c}_{1}^{2}$, where *c*_1_ and *c*_2_ are the concentrations of monomers and dimers, respectively. We first note that the concentration 200 nM is higher than the cmc (≈120 nM). This means that at the cmc, monomers are the relevant subunits that self-assemble into micelles. Then, above the cmc, *c*_1_ is expected to vary only weakly with increasing total concentration. If *c*_1_ is approximately constant, then it follows that also ${c}_{2}={K}_{2}{c}_{1}^{2}\approx 0$ is approximately constant, and hence we do not expect any significant dimerization at higher concentrations either.

When measuring the equilibrium micelle size distribution by mass photometry, some aspects need to be considered. Firstly, the dissociation of micelles upon dilution to the nM range (allowing for single particle measurements) needs to be much slower than the total measurement time (typically 60 s), otherwise the data will not represent the equilibrium size distribution at the initial concentration. This condition is met in the case of JB6 at ambient temperature (22 °C), where the dissociation takes place on the time scale of tens of hours. Secondly, it is important to use a set of calibration proteins with the same polarizability as the analyte. Here, it is verified that the *dn*/*dc* is similar for the globularly folded BSA and the large self-assemblies of JB6. This is not surprising, since the refractive index of proteins has been found to be highly similar^36,52^, but the confirmation is nevertheless important in order to trust the masses obtained.

A similar concern regarding micelle dissociation applies to the AUC experiment. The centrifugation partly changes local concentrations, altering the micelle size distribution. However, when dissociation is slow, as here, this is not an issue, and AUC reports on the equilibrium size distribution. The fact that the mass photometry and AUC distributions are in strong agreement (Figs. 3, 5, and 7) confirms that that we are in the slow dissociation regime.

A broad JB6 micelle size distribution has been reported previously^4,21,22^. Here, however, we were able to quantify the full size distribution and moreover demonstrate how it varies with the concentration (Fig. 1) as well as with solution conditions such as temperature (Fig. 3), pH (Fig. 5), and added electrolyte (Fig. 8).

From the obtained mass photometry size distribution, the mean aggregation number, 〈*N*〉, is readily calculated as a useful quantitative parameter to characterize e.g. the concentration dependence. Varying the concentration from 1 to 100 μM, 〈*N*〉 was found to increase by a factor of 2, from ca. 20−40. The distribution is characterized by a broad maximum that shifts to higher *N* with increasing concentration. We note that small aggregation numbers are unstable (low frequency of observation). This is most likely because they do not sufficiently hide hydrophobic residues from water, similar to the case of surfactant micelles^23^.

The size distributions at 22 and 37 °C are strikingly similar, but the dissociation rate depends strongly on temperature. We therefore conclude that the energy landscape is similar over the temperature interval, with a thermally activated process separating the micelles and monomers. From the Arrhenius plot (covering 22−40 °C) we obtain an activation barrier of ~250 kJ/mol. This is a comparable barrier to other reported activation barriers for protein complex dissociation, such as high-density lipoprotein dissociation with a 209 kJ/mol barrier^53^, very-low-density lipoprotein dissociation with two 222 kJ/mol barriers^54^, and *α*B-crystallin with 79.5−251 kJ/mol^55,56^, whereas a somewhat lower, 99 kJ/mol, barrier was derived for the dissociation of the TIM barrel dimer of two 26 kDa chains^57^. In addition, the dissociation kinetics reveal that the micelles dissociate on the time scale of 20 min at 37 °C. Taking into account that the active species of JB6 are the monomers and not the micelles^29^, we can now understand why the activity of JB6 is high in amyloid aggregation kinetic experiments at 37 °C when the lag phase is longer than about 30 min. The observed difference in 〈R_H_〉 after dissociation at different temperatures can be explained with the mass photometry data at 22 and 37 °C, where it is evident that a larger part of the micelles are kept from dissociation at the lower temperature.

The micelle size distributions at 30 μM JB6 were compared at different pH values (Fig. 5). At pH 5.0, 7.4, and 8.0, there is a very similar, broad micelle size distribution with 〈*N*〉 ≈ 35. However, interestingly, between pH 5 and 4 there is a sharp transition to a more narrow distribution and 〈*N*〉 ≈ 10. The shift in size distribution coincides with the denaturation of the CTD, as evaluated with CD spectroscopy. Clearly, by denaturating the CTD, the packing of protein molecules in the micelles is significantly altered. The CTD being less stable compared to the NTD agrees with the findings in^42^, where the CTD was found to have both lower chemical and thermal stability than the NTD. While there is a large (a factor of 3–4) change in 〈*N*〉, there is only a small change in 〈R_H_〉 at the transition (Fig. 5b). This can be explained by the disordered CTD at low pH values. When a part of the protein is a random coil, it greatly expands the effective protein size, which is also reflected in the large water fraction of these micelles (Fig. 7h). Considering that micelles are formed also at the extreme condition of pH 2, with the CTD unfolded and a protein net charge of +40, we can conclude that the main driving force for micelle formation is not electrostatic, and does not require a folded CTD. Instead, it is most likely hydrophobic interactions in the linker region, which are responsible for the strong self-assembly. This is further supported by the low self-affinity observed for the isolated CTD^15^. Nevertheless, at physiological pH values there may be synergistic effects between the linker and CTD and the dramatic shift in size distribution upon unfolding of the CTD is in line with findings from^58^ and^59^ that the CTD:s are in close proximity in the micelle state. Based on these reports and the current data, we speculate that the interaction sites at different positions of the protein may enable a more flexible micelle structure, enabling a wider interval of aggregation numbers. This would explain the broad size distribution of JB6 at pH values where the CTD is folded, as well as the more narrow distribution when it is unfolded.

Upon addition of 1:1 electrolytes (150 mM sodium salts), specific anion effects on the micelle size distribution were observed, in accordance with the Hofmeister series^47,48^. For typical salting out anions (fluoride and acetate) essentially no effects on the micelle size distribution was observed. However, the addition of typical salting in anions (iodine and thiocyanate) resulted in a decrease of the micellar size. This is likely the effect of an increased electrostatic repulsion in the micelles due to anion adsorption at hydrophobic patches^32,50^. Chloride, which is a border-line ion in the Hofmeister series^48^, here behaved as a weak salting-in ion. A similar decrease in micellar size was also observed with chloride, but at a higher salt concentrations (≈500 mM) and the micelles remained stable even at 1.3 M salt. That chloride behaves as a salting in ion is consistent with the report of specific adsorption of chloride to a model protein (lysozyme)^60^.

## Methods

First, general methods of all experiments and techniques are described, followed by methods specific to each experiment.

### JB6 expression and purification

Human DNAJB6b was expressed, purified, and fluorescently labeled as described in a previously published protocol^61^. The protein is found to be highly pure (Supplementary Note 2, Fig. S2). The fluorescently labeled JB6 was Alexa647 (Thermo Fisher Scientific) coupled to an added cysteine at the N-terminus of JB6 (this labeling position has minimal influence on the self-assembly, as investigated in Supplementary Note 8, Fig. S8). The CTD (residue number 186-241) was expressed and purified as described in ref. ^15^ and the NTD (residue number 1-71) as described in^42^. ΔST JB6 (with the deletion of residues 132-184 from wt) was expressed and purified as described in Supplementary Note 7, Fig. S7.

### Sample preparation

The standard buffer used in all cases was 20 mM NaP, 0.2 mM EDTA, pH 8.0 with additions to obtain the solution conditions examined, filtered through a wwPTFE-filter with 0.22 μm pore size. In the cases where equilibrium size distributions were examined, the samples were equilibrated at room temperature (21–22 °C), for 6–8 days for all samples except for the one at 37 °C which was measured after 5−30 h. These equilibration times were chosen based on the dissociation kinetics found in this work, Fig. 4.

In the case of the 463 μM JB6 sample used in the mass photometry and refractive index measurements, the high concentration was achieved by lyophilizing several fractions of JB6 and dissolved in 6 M GuHCl, 20 mM NaP, pH 8.0. Dialysis with a Slid-A-Lyzer MINI Dialysis, 3.5 K MWCO, 2 ml, was done to change buffer to 20 mM NaP, 0.2 mM EDTA, pH 8.0. This was done by changing buffer solution (45 ml to 0.5 ml sample) every hour, with 100 rpm shaking in between, 5 times, and then again after 24 h. The conductivity was measured of all discarded buffers, see Supplementary Note 3, Fig. S3.

### Analytical ultracentrifugation, AUC

An Optima AUC (Beckman Coulter Inc.) was used in velocity sedimentation mode, at 40,000 rpm, with an An-60 Ti rotor with epon centerpieces and quartz glass windows. 140 absorbance scans at 280 nm were collected during a run time of 148 min, at 20 or 37 °C. The software Ultra Scan 3 was used for experimental design and analysis, following the 2DSA data analysis as described in ref. ^51^ and ending with a Monte Carlo analysis (50 iterations). The absorbance scans to all experimental results shown is included in the SI, Figs. S10–12.

### Mass photometry

The instrument TwoMP (Refeyn Ltd., Oxford, UK) was used in “buffer-free” mode (20 μl of the sample was used for each measurement), in regular windows size, data collection during 1 min, at ambient temperature (22–24 °C), for all measurements. The samples of JB6 concentrations >200 nM were diluted to 200 nM less than a minute before measurement. Monomer and dimer peaks of IgG, BSA, and thyroglobulin were used for calibration, diluted to ca. 10 nM each, in 20 mM NaP, 0.2 mM EDTA, pH 8.0 (more information in Supplementary Note 1, Fig. S1). diS100G, ΔST JB6, sfGFP, crimson, and calbindin D_28k_ (all five Cys mutated to Ser, to avoid intermolecular disulfide bonds) were recombinantly produced, with amino acid sequences given in Supplementary note 10. Ovalbumin, BSA, and thyroglobulin were purchased from Cytiva, IgG from Lee BioSolutions, obtained as powder products. In all mass photometry histograms of JB6, the binsize is set to 26.9 kDa, the molecular weight of JB6. The two exceptions are in Fig. 1b and e, where the binsize is 2 kDa for all proteins. To study the masses in the 30−40 kDa regime, the buffer was filtered through a 5 kDa cutoff spin-filter (Vivaspin 20, Startorius, 5000 MWCO PES). This reduced the buffer contribution to about 10 counts, which is negligible.

### Microfludic diffusional sizing, MDS

The instrument Fluidity One M (Fluidic Sciences, Cambridge, UK) was used in size setting 3 and viscosity setting 1 if not else is stated. At least triplicates of all data points were obtained at ambient temperature (22–23 °C). The samples were incubated in dark to reach equilibrium. For the ΔST JB6, a Fluidity One instrument (Fluidic Analytics, Cambridge UK) was used, which required no labeled protein.

### Dissociation kinetics of JB6

For the dissociation kinetic measurements with MDS, Alexa647-JB6 (62 % labeling efficiency, see Supplementary Note 9 Fig. S9) was diluted in thermally equilibrated buffer at time zero from 5.8 μM to 55 nM. Final volume was 2 ml in a 5 ml Eppendorf protein low binding tube (to minimize loss of protein due to surface adsorption). During thermal incubation in dark, a few μl were used to obtain 〈R_H_〉 as a function of time. Size setting 3 was used for 〈R_H_〉 above 6 nm, setting 2 for below, in at least triplicates.

Similar procedure was performed with the mass photometer, but with non-labeled protein, a final concentration of 50 nM, and a volume of 5 ml. To obtain the oligomeric mass, the sum of all counts times each mass (mass weighted) of *N* > 2 was used. Supplementary Note 6, Fig. S6 shows that surface adsorption is negligible (ca. 10 %) in this setup.

### Light scattering

Precipitation phase boundaries of JB6, in the pH range 2.9-8.0, were investigated at room temperature using light scattering intensity of 1 ml of 10 μM JB6 in a Labbot (Labbot, Lund, Sweden), 638 nm laser, at 90 degrees scattering angle. A 2 min waiting time was used between additions of 5−10 μL of 20 mM phosphoric acid to change pH from the initial 8.0 down to 2.9 (keeping the phosphor ion concentration constant at 20 mM). An Orion Star A211 pH meter was used to measure pH.

### Circular Dichroism (CD) spectroscopy

Fractions of the same JB6 samples at different pH values were diluted from 30 μM to 5 μM in the same buffer at the same pH, prior to measurements with CD spectroscopy (J-815, JASCO). A quartz cuvette with 1 mm pathlength was used at 20 °C (adjusted with a Peltier temperature controller), 250−185 nm UV range, 50 nm/min, D.I.T. 8 s, 1 nm bandwidth, average of 3 accumulations.

### Refractive index measurements

JB6 at 12.5 mg/ml (463 μM) and BSA at 14.5 mg/ml were diluted in series with about a factor of 1.5, down to 0.1 mg/ml. 50 μL of each concentration were used in a manual Abbe refractometer (Bellingham + Stanley) to determine the refractive index at 546 nm and 579 nm. The temperature was controlled and kept at 20 °C.

### Temperature variation

The micelle formation at 37 °C was compared to room temperature using AUC and mass photometry. Fractions of the same sample (30 μM JB6 in 20, mM NaP buffer, 0.2 mM EDTA, pH 8.0) were used for measurements with both AUC and mass photometry in parallel. The samples had been let to equilibrate after preparation for 6 days at room temperature, or 37 °C for at least 5 h, respectively.

### pH variation

Phosphoric acid was used to adjust the pH of 20 mM NaP buffer (initially pH 8.0), 0.2 mM EDTA, at room temperature. The same amount of phosphoric acid needed to reach pH 2.0, 3.0, 4.0, 5.0, 7.4, 8.0 was then used to adjust the protein stock (same buffer) in a one step addition to the required pH. The pH was checked of the final samples after dilution with each pH-adjusted buffer, to 30 μM JB6 and 20 nM Alexa647-JB6. After equilibration at room temperature for 6 days, the samples were analyzed with MDS, mass photometry, AUC, and CD spectroscopy. For the CD spectroscopy, the samples were diluted to 5 μM in the same buffer at the same pH prior to measurements.

### Ionic strength

JB6 in 20 mM NaP, 0.2 mM EDTA, pH 8.0, at room temperature, was mixed with 5 M NaCl in the same buffer to final concentrations of 0-1.25 M NaCl, 5 μM JB6, and 20 mM Alexa647-JB6. Measurements with MDS and mass photometry were performed after 6 days incubation at room temperature. The mass photometer was calibrated using the same salt concentration and buffer as the samples.

### Salts spanning the Hofmeister series

Solutions of 150 mM NaF, NaOAc, NaCl, NaI, and NaSCN, respectively, were prepared in 20 mM NaP buffer, 0.2 mM EDTA, pH 8.0. 30 μM JB6 solutions with respective salt were prepared and spiked with 20 nM Alexa-labeled JB6. The samples were let to equilibrate for 6 days at room temperature before parallel measurements with MDS and AUC.

### Reporting summary

Further information on research design is available in the Nature Portfolio Reporting Summary linked to this article.

## Supplementary information

Supplementary Information

Reporting summary

## Data Availability

Data that support the findings of this study have been deposited in https://github.com/saralinse/Published_Data/tree/CommChem_2025_JB6_conditions.
