# Supplementary material for: Effects of solution conditions on the self-assembly of the chaperone protein DNAJB6b
Source: Commun Chem. 2025 Oct 1;8:289. doi: 10.1038/s42004-025-01697-7 (PMC12488921; doi:10.1038/s42004-025-01697-7)
Supplement: Supplementary file 1 — Supplementary Information [file 42004_2025_1697_MOESM1_ESM.pdf]

Supplementary Information to

Effects of solution conditions on the self-assembly of the chaperone protein DNAJB6b

Andreas Carlsson[1*], Victoria Maier[1,2], Celia Fricke[1,3], Tinna Pálmadóttir[1], Ingemar André[1], Ulf Olsson[4] and Sara Linse[1]

[1] Biochemistry and Structural Biology, Department of Chemistry, Lund University, SE-221 00 Lund, Sweden
[2]Center for Functional Protein Assemblies and Department of Bioscience, TUM School of Natural Sciences, Technical University of Munich (TUM), 85748 Garching, Germany, Germany (current affiliation)
[3] Department of Biotechnology and Biomedicine, Technical University of Denmark, 2800 Kongens Lyngby, Denmark
[4] Division of Physical Chemistry, Department of Chemistry, Lund University, SE-221 00 Lund, Sweden
*Andreas Carlsson, E-mail: andreas.carlsson@biochemistry.lu.se

Supplementary note 1: Mass photometry calibration

The mass photometer was calibrated using human IgG, BSA, and bovine thyroglobulin, both monomer and dimer peaks, as demonstrated in Figure S1.

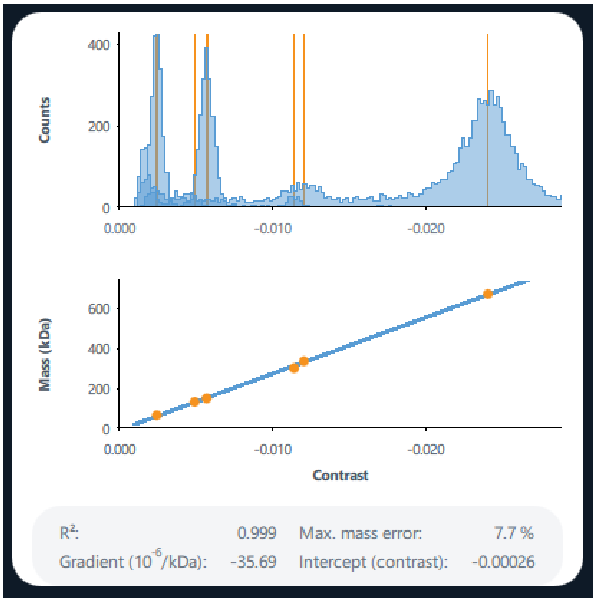

Figure S1: Calibration used for the mass photometer measurements. Monomer and dimer peak of all the three proteins BSA, IgG, and thyroglobulin.

Supplementary note 2: Purity of JB6

The purity of JB6 with the used purification protocol was demonstrated in (1). To further examine the purity, HPLC was used, measuring the absorbance at 205 nm to estimate the level of any impurities. As seen in Figure S2, the concentrations of any impurities are too low to be seen on the out-zoomed chromatogram, or in an SDS PAGE with a heavy load of JB6 (7 µL of 30 µM). When zooming in on the HPLC chromatogram, two to four small peaks can be seen. They have the peak area of about 0.1 % or less of the JB6 peak. We conclude that the JB6 sample has a purity level of $>$ 99.5 %. For the HPLC, a C18 reversed phase column (BIOshell A160 Peptide CN column 66966-U, Sigma-Aldrich) was used. The column was operated at 60 °C in an aqueous mobile phase with 0.1 % TFA, at 0.5 ml/min. Elution during 10 minutes with a linear gradient from 5 to 95 % acetonitrile.

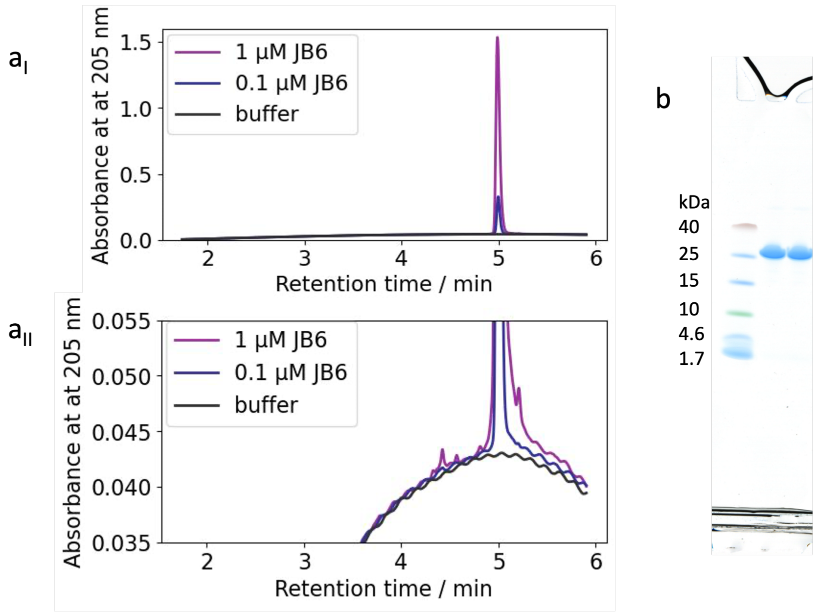

Figure S2: **a**: UV absorbance at 205 nm in reversed phase on HPLC. a_I shows whole chromatograms and a_{II} in-zoom on eventual impurities (of the size of 0.1 % of the JB6 peak). **b**: SDS PAGE of two lanes with 30 µM JB6.

Supplementary note 3: Dialysis of 463 µM JB6

To obtain a JB6 concentration of 463 µM (the highest concentration used for mass photometry and refractive index measurements, Figure 1 in the main article), several aliquots of about 20-50 µM were lyophilized, dissolved in 6 M GuHCl of about 1/20 of the initial volume, pooled into a volume of 0.5 ml and then dialyzed with a Slid-A-Lyzer MINI Dialysis, 3.5 K MWCO, 2 ml. 6 changes of 45 mL of 20 mM NaP, 0.2 mM EDTA, pH 8.0, changed every hour, with 100 rpm shaking in between, and the last incubation lasted for 24 h. The conductivity was measured on all discarded buffers, plotted in Figure S3.

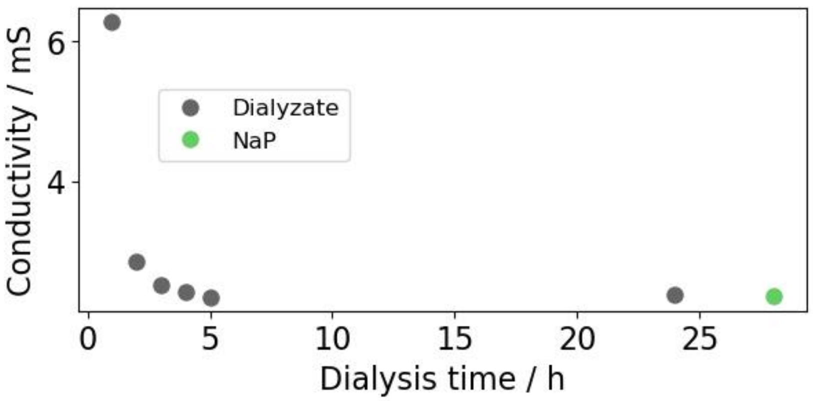

Figure S3: Conductivity measurements of the six dialysates to obtain 463 µM JB6.

Supplementary note 4: JB6 as a monomer at different conditions

From Figure 2 in the main article, it is evident that JB6 is monomeric at 10 nM. To understand if the subunits are monomers also at higher concentrations, and if a higher salt concentration and pH 7.4 has any influence, some different samples are compared in Figure S4. In panel a, JB6 is observed as monomers in all samples. Panel b shows that the monomers is in equilibrium with the micelles at higher protein concentrations. The samples were equilibrated as described in the Methods section of the main article. Less than a minute before measurements, the 5 and 34 µM samples were diluted to 200 nM.

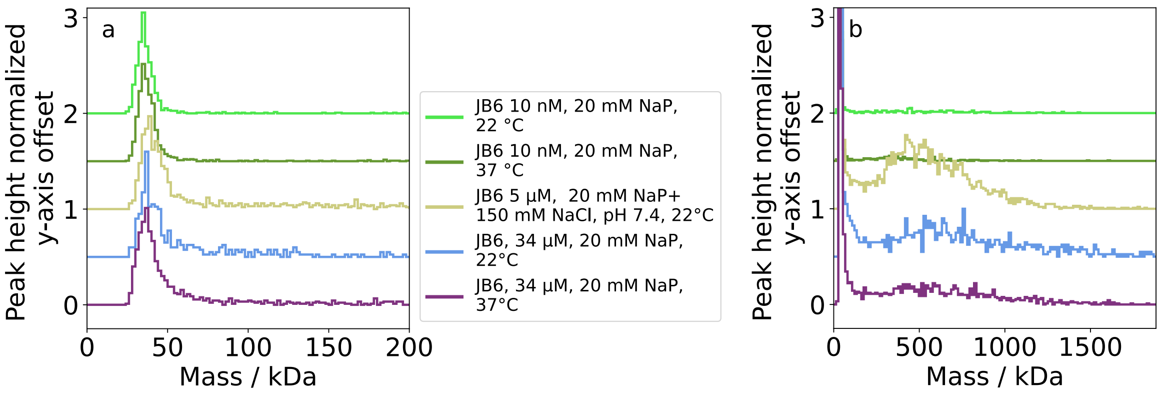

Figure S4: Comparison of different sample conditions. Low mass range in **a** of some different samples with JB6, and larger mass range in **b**. The bin size is 2 kDa in panel a and 10 kDa in panel b.

Supplementary note 5: Conversion to mass-weighted distributions of mass photometry data

The raw data from mass photometry is histograms with the number of particle counts detected for each scattering intensity of the binning, which is converted to mass using a calibration. This corresponds to the number weighted distribution in the case of a self-assembly system. To compare with a mass-weighted distribution, such as obtained from the absorbance quantification in the AUC, the number weighed distribution can be multiplied with the aggregation numbers, N, i.e. the detected mass divided by the molecular weight of JB6. Both the counts and the counts*N distributions of 55 µM is shown in Figure S5.

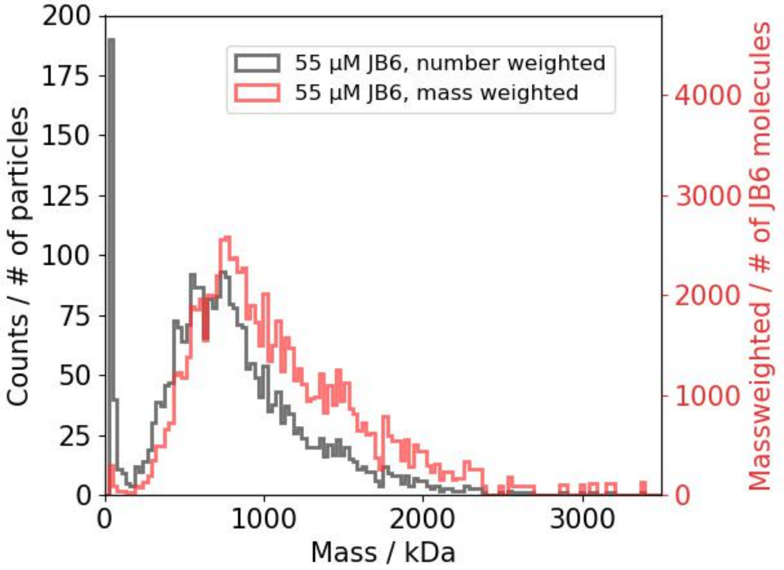

Figure S5: Mass photometry data of 55 µM, plotted with both number weighted (counts) distribution and mass weighted.

Supplementary note 6: Surface adsorption in 5 ml low binding tubes

The parameter used to follow the micelle dissociation with mass photometry (micelle mass * $\langle N \rangle$), is sensitive to concentration changes due to surface adsorption. To learn whether this is a significant problem at the conditions used, the concentration was measured as a function of time since dilution. A step dilution was made from 34 µM to 50 nM respectively 20 nM in a volume of 5 ml, in a 5 ml protein low binding Eppendorf tube. HPLC in reversed phase (see operating details in the section above "Purity of JB6") was used to measure the concentration by absorbance at 280 nm. To minimize the surface adsorption in the HPLC, each injection was done directly after adding 100 µM of the sample in a PEGylated 96 well half-area plate. The theoretical extinction coefficient of 14440 cm^{-1} M^{-1} was used to obtain the concentration. The deviation from the aimed 20 and 50 nM (17.2 and 41 nM) is not unreasonable considering the different quantification methods, pipetting error of the heavy dilution, and eventual sample losses in the HPLC. What is important is the relative concentration difference in the same sample, which is subject to the same treatment. An exponential decay function was used to fit respective series. At 50 nM, 4.0 nM of the initial 41 nM, about 10 %, is lost in surface adsorption. At 20 nM the loss is 3.4 nM of initial 17.2 nM, about 20 %, which is a reason to choose 50 nM to work with. Since the parameter used in Figure 4B decreases from more than 600 GDa to about 100 GDa, surface adsorption of 10 % is neglected in the analysis.

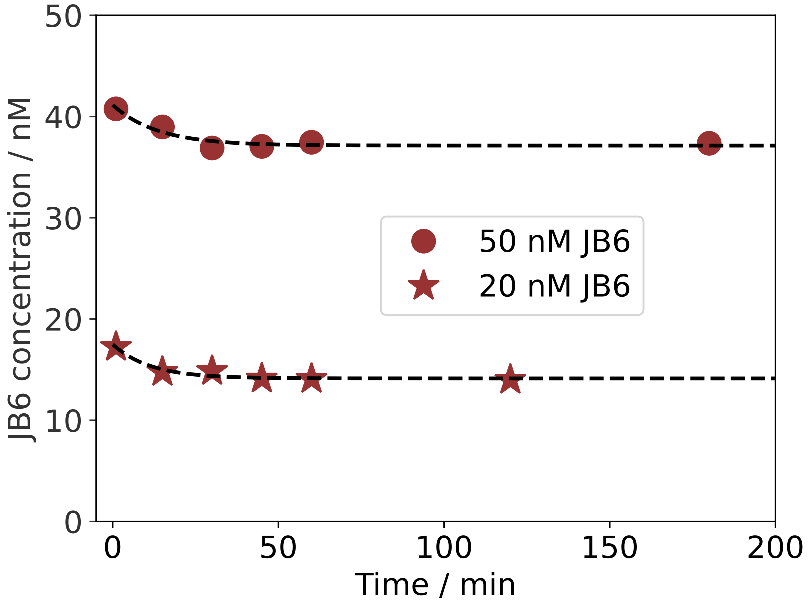

Figure S6: Surface adsorption of JB6 in 5 ml Eppendorf protein low binding tubes, measured with HPLC, absorbance at 280 nm. Fits to exponential decay functions on the form $y(t) = a * \mathrm{e}^{-bt} + c$ in dotted lines.

Supplementary note 7: ΔST JB6 expression and purification.

The ΔST JB6 variant was expressed in the same way as the wt JB6, in *E. coli* BL21 DE3 pLysS*, described in (1). The sequence is given in black in Figure S7a. The deleted part, residue 132-184 is located in the flexible linker region. The purification was performed in the same way as for wt JB6, as described in (1), with the exception that 37 % of saturated ammonium sulfate was used to precipitate the protein (Figure S7b shows the supernatant of 15, 22, and 37 % ammonium sulfate solutions after centrifugation), compared to 22 % for wt JB6. After size exclusion in a Superdex 200 column with 2 M guanidine hydrochloride as running buffer (20 mM NaP, 0.2 mM EDTA, pH 8.0), fraction B5 and B6 (panel c) were pooled, lyophilized, dissolved with water to 5 ml, and run on a Superose 6 column, with 20 mM NaP, 0.2 mM EDTA, pH 8.0 as running buffer. The C1 fraction (panel d) was used for the experiments in this work, which had a concentration of 55 µM as determined with absorbance spectrum and theoretical extinction coefficient of 14440 cm^{-1} M^{-1}.

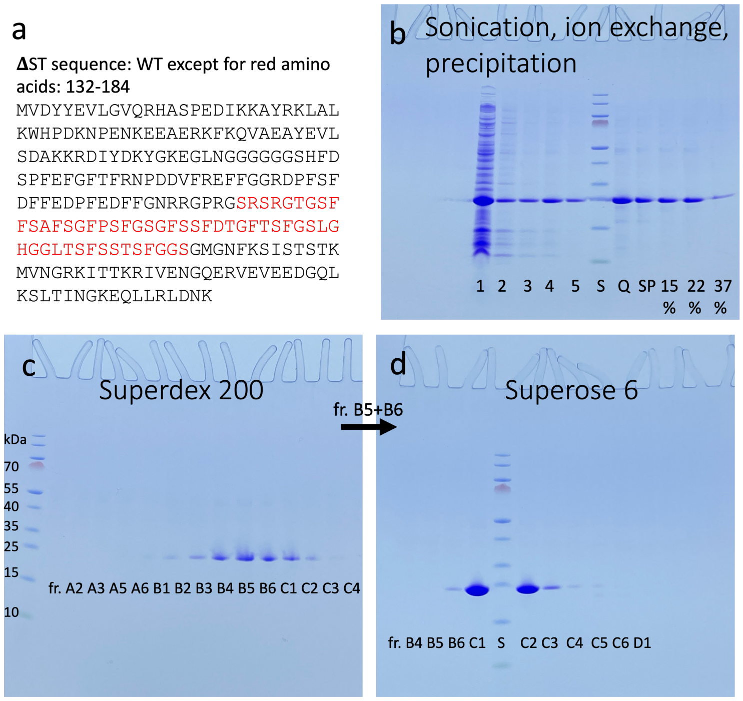

Figure S7: ΔST JB6 purification. **a**: Sequence of ΔST JB6 in black, compared to the whole, wt JB6 sequence. **b-d**: SDS PAGEs of different parts of the purification. **b**: Lane 1-5 are sonicates 1-5. S is a standard (Thermo Fisher pre-stained ladder). Q is the flow-through from Q-resin anionic exchange. SP is the flow-through from SP-resin catatonic exchange. Lanes marked 15, 22, 37 % contains the supernatant after centrifugation with 15, 22, and 37 % of saturated ammonium sulfate solutions. **c**: SEC on Superdex 200 of the dissolved pellet after ammonium sulfate precipitation, in 2 M guanidine hydrochloride 20 mM NaP, 0.2 mM EDTA, pH 8.0 as running buffer. **d**: SEC on Superose 6, with 20 mM NaP, 0.2 mM EDTA, pH 8.0 as running buffer.

Supplementary note 8: Can we label JB6 with a fluorophore without disturbing its self-assembly?

To investigate the extent to which the fluorophore affects the self-assembly of JB6, the average hydrodynamic radius, $\langle R_H \rangle$, was measured as a function of the fraction labeled protein. This was done for both N-cys and C-cys JB6, with the total protein concentration kept at 2 μM. The samples were let to equilibrate at room temperature, in the dark, for 7 days before measurements with MDS (Fluidity One M), using size setting 3, in four technical replicates. Since the N-cys stock solution had a 62 % labeling efficiency, this is the highest fraction measured for N-cys.

From the $\langle R_H \rangle$, it is observed that C-cys JB6 is strongly influenced by the fraction labeled protein above 10 %, but not at lower ratios. N-cys seems to be less affected in its self-assembly up to 62 %. This is supported in the total fluorescence intensity (which is recorded for each measurement), since the C-cys deviates from a straight line at higher labeling fractions than about 20 %, possible due to self-quenching of fluorophores in close proximity. This is not the case for N-cys, where no self-quenching is observed up to the highest ratio of 62 %. The potentially smaller disturbance on the self-assembly of N-cys is the reason we choose this labeling position.

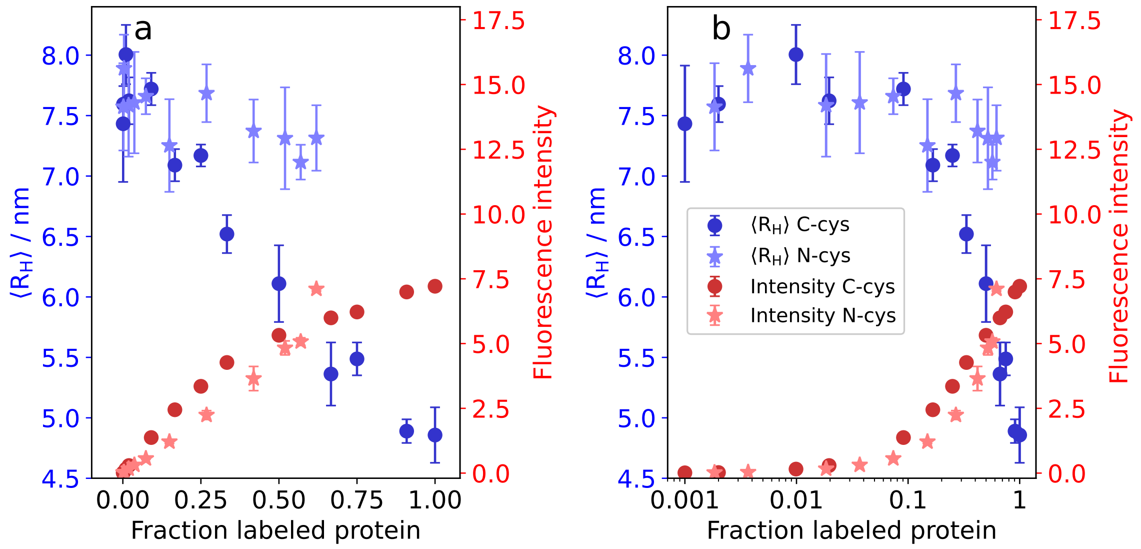

Figure S8: Labeling effect on JB6 self-association. 2 μM total JB6 concentration and varying fraction labeled JB6, both with JB6 labeled at the C-terminal (C-cyc) and N-terminal (N-cys). Using MDS, both $\langle R_H \rangle$ and fluorescence intensity is measured and plotted to the left respectively right y-axis, towards **a** linear and **b** logarithmic x-axis.

Supplementary note 9: Labeling efficiency

The labeling efficiency was determined using absorbance spectra and the known extinction coefficients for JB6 and the fluorophore (ϵ_{280}, JB6 = 14440 cm^{-1} M^{-1}, ϵ_{650}, Alexa647 = 265 000 cm^{-1} M^{-1}, and ϵ_{280}, Alexa647 = 7950 cm^{-1} M^{-1}). C-cys was estimated to close to 100 % labeled. N-cys was estimated to 62 %, using abs$_{280}$ = 0.0225 when subtracting the estimated baseline. The Alexa647 concentration is 0.19/265000 = 0.717 μM, providing a contribution to the abs$_{280}$ of 7950*0.19/265000 = 0.0057. The protein contribution to the abs$_{280}$ is 0.0225-0.0057 = 0.0168, providing a concentration of 0.0168/14440 = 1.16 μM. Hence, the labeling efficiency is 0.717/1.16 = 62 %.

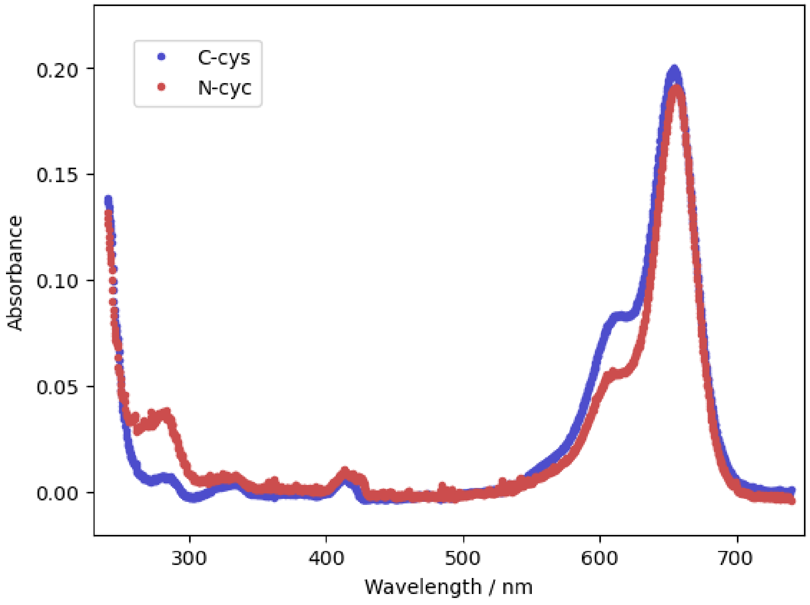

Figure S9: Labeling efficiency determination of Alexa647-JB6, using absorbance spectroscopy of both C-cys and N-cys JB6.

Supplementary note 10: Sequences of diS100G, ΔST JB6, GFP, crimson, and calbindin D_{28k}

These are the recombinantly produced proteins measured with the mass photometer. Their respective amino acid sequence is given below.

diS100G: MKSPEELKRIFEKYAAKEGDPDQLSKDELKLLIQAEFPSLLKGMSTLDDLFQ ELDKDGDGEVSFEEFQVLVKKISQGRSPEELKRIFEKYAAKEGDPDQLSKDELKLLIQ AEFPSLLKGMSTLDDLFQELDKDGDGEVSFEEFQVLVKKISQ

ΔST JB6: MVDYYEVLGVQRHASPEDIKKAYRKLALKWHPDKNPENKEEAERKFKQ VAEAYEVLSDAKKRDIYDKYGKEGLNGGGGGGSHFDSPFEFGFTFRNPDDVFREFFG GRDPFSFDFFEDPFEDFFGNRRGPRGGMGNFKSISTSTKMVNGRKITTKRIVENGQE RVEVEEDGQLKSLTINGKEQLLRLDNK

sfGFP: MSKGEELFTGVVPILVELDGDVNGHKFSVRGEGEGDATNGKLTLKFICTTGK LPVPWPTLVTTLTYGVQCFSRYPDHMKRHDFFKSAMPEGYVQERTISFKDDGTYKT RAEVKFEGDTLVNRIELKGIDFKEDGNILGHKLEYNFNSHNVYITADKQKNGIKANFK IRHNVEDGSVQLADHYQQNTPIGDGPVLLPDNHYLSTQSVLSKDPNEKRDHMVLLEF VTAAGITHGMDELYKG

crimson: MVSKGEELIKENMRSKLYLEGSVNGHQFKCTHEGEGKPYEGTQTNRIKVVE GGPLPFAFDILATMFMYGSKAFIKYPKGLPDYFKQSFPEGFTWERTMVFEDGGVLT ATQDTSLQDGELIYNVKLRGVNFPANGPVMKQTTLGWEPSTETLYPADGALEGRCD MALKLVGGGHLHCNFKTTYKSKKPVKMPGVHYVDRRLERIKEADNETYVEQHEVA VARYCDLPSKLGHKLNGMDELYK

calbindin D_{28k} (all five Cys mutated to Ser, to avoid intermolecular disulfide bonds): MAE SHLQSSLITASQFFEIWLHFDADGSGYLEGKELQNLIQELQQARKKAGLELSPEMKTFV

DQYGQRDDGKIGIVELAHVLPTEENFLLLFRSQQLKSSEEFMKTWRKYDTDHSGFIET
EELKNFLKDLLEKANKTVDDTKLAEYTDLMLKLFDSNNDGKLELTEMARLLPVQENF
LLKFQGIKMSGKEFNKAFELYDQDGNGYIDENELDALLKDLSEKNKQDLDINNITTYK
KNIMALSDGGKLYRTDLALILSAGDN

AUC absorbance scans

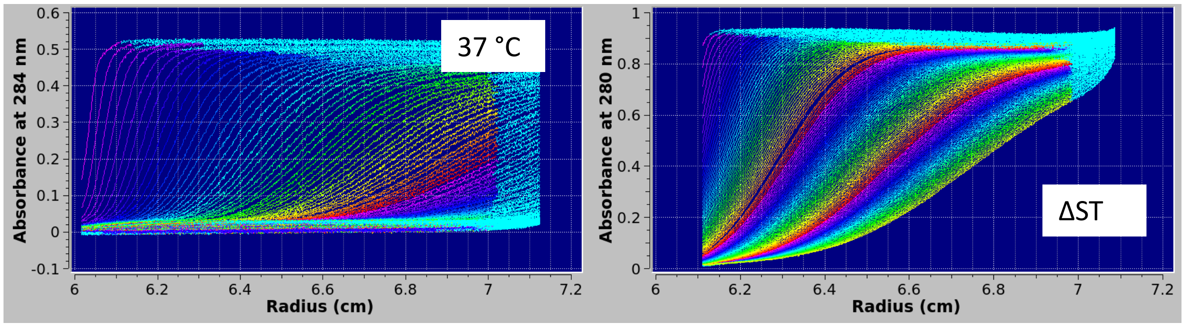

Figure S10: AUC absorbance scans after noise reduction of 30 μM JB6 at 37 °C and the ΔST JB6 (at room temperature), 55 μM.

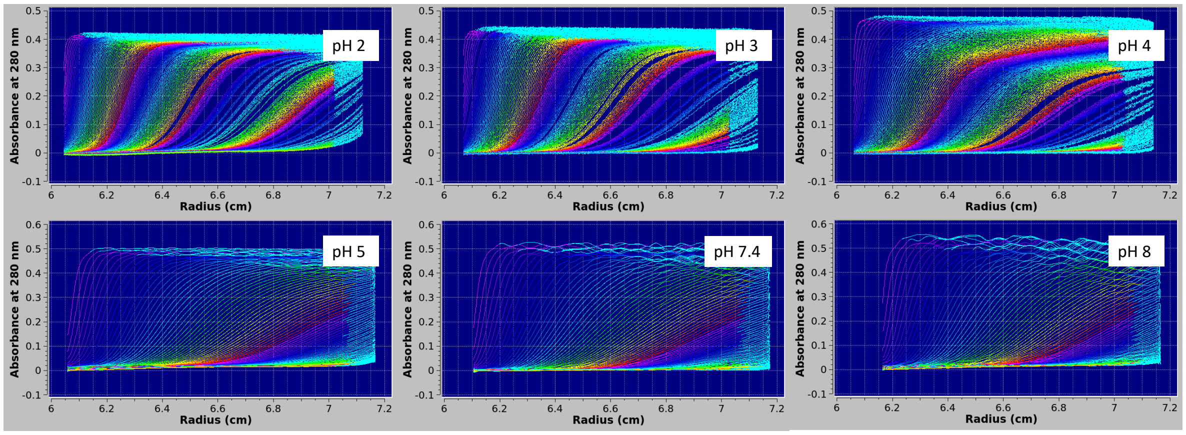

Figure S11: AUC absorbance scans after noise reduction of 30 μM JB6 at different pH values.

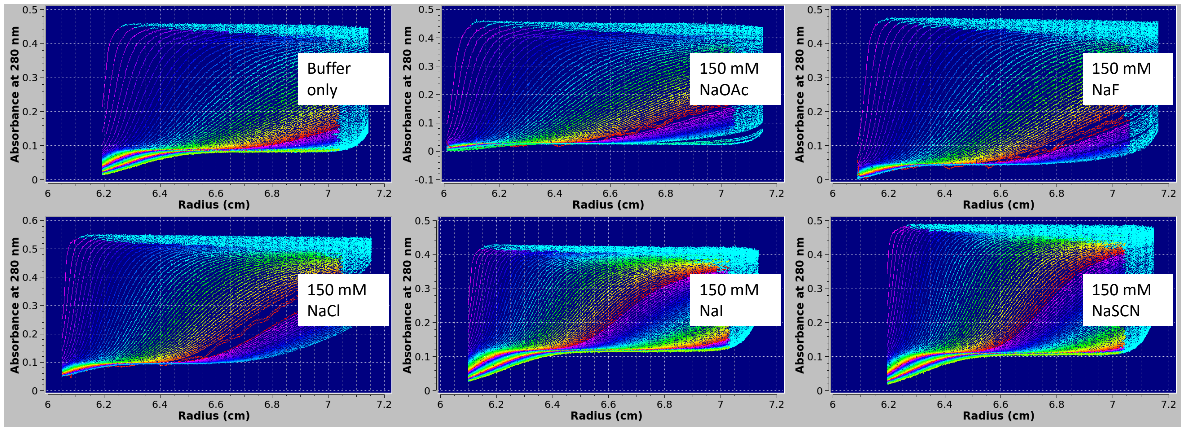

Figure S12: AUC absorbance scans after noise reduction of 30 μM JB6 in 150 mM salts across the Hofmeister series.

References

[1] Linse, S. High-Efficiency Expression and Purification of DNAJB6b Based on the pH-Modulation of Solubility and Denaturant-Modulation of Size. *Molecules* **27** (2022).
